# Twelve tips for UK medical students undertaking laboratory-based intercalated research projects

**DOI:** 10.15694/mep.2020.000225.2

**Published:** 2021-09-03

**Authors:** Lydia Roberts

**Affiliations:** 1 School of Clinical Medicine; 2 School of Clinical Medicine

**Keywords:** Intercalation, research, undergraduate, medicine, study skills, basic sciences

## Abstract

This article was migrated. The article was marked as recommended.

Laboratory-based intercalated research projects are a popular undertaking for medical students in the UK. Such projects can provide students with a wealth of valuable experiences and the chance to develop new skills that will be highly beneficial to their future careers. Laboratory-based intercalated research projects however represent a very different challenge to other aspects of medical education, with distinct expectations and requirements of students in order for success to be achieved. In this article, the author compiled twelve tips based on current literature and their experiences carrying out a laboratory-based intercalated research project as a UK medical student. These tips will help ensure UK medical students are well prepared before commencing their intercalated research project, in order to maximise the benefits of the opportunities presented to them. Although focused on a UK perspective, many of the tips will similarly be applicable to medical students in other countries conducting laboratory-based projects, albeit in slightly different contexts.

## Introduction

An intercalated degree is an additional undergraduate degree that may be completed by medical students in a year taken out from their standard medical curriculum. Students can choose to pursue an intercalated degree in any subject of their choosing, however most frequently students choose a biological science discipline, with the completion of a laboratory research project as part of the course being a popular option ( Leung, 2001). Intercalation is a compulsory component of a growing number of UK six-year medical degree programmes, including the Universities of Cambridge, Oxford, and Imperial College London. At other institutions, medical students have the option to intercalate at different points during their medical training and may even decide to apply to study on an intercalated degree programme at a different university ( Hull York Medical School, 2019).

Intercalated degree programmes offer many benefits to students, providing extensive opportunities to develop skills in critical scientific thinking, evidence evaluation and academic writing ( Philip, Prasad and Patel, 2015). Practical laboratory projects also enable students to acquire knowledge and experience in scientific research techniques. These experiences provide a great foundation for students interested in pursuing a career in clinical academia ( Morrison, 2004), but can also be advantageous more generally. For example, medical students are currently awarded additional points on their Foundation Programme Application for holding additional degrees ( UK Foundation Programme, 2020).

Despite their merits, intercalated degree programmes are not without their disadvantages. These include extending the cost of studying and delaying graduation by a year, on what is already a lengthy degree programme ( Nicholson *et al.*, 2010). In addition, the intercalated degree programme can itself be challenging and a source of considerable stress and anxiety. In particular, if students undertake a laboratory research project as part of their intercalated degree, the nature of their work and study will contrast starkly with the previous years of their medical education ( Siemens *et al.*, 2010). This can be very daunting, with students expected to make significant adjustments to their previous approach to studying. The intercalated degree is only a year long, with actual laboratory time being shorter than this, so students have little time to work out the best strategy to take. This can leave students frustrated come the end of their research project, as they feel they failed to use their time in the most effective manner.

In this article, twelve tips are presented on how to get the most out of a laboratory-based intercalated research project. These are based on the present literature and the author’s own personal experiences of carrying out an intercalated research project as a part of their medical degree programme in the UK.

## Twelve Tips

### Tip 1: ensure intercalation is for you

Intercalation can provide medical students with a wealth of valuable opportunities, enabling them to explore a topic in more depth than would normally be possible as part of their medical degree and develop additional skills that will complement their clinical practice ( BMA, 2020). The decision to intercalate however is not one that should be taken lightly. UK medical degree courses are already a minimum of five years, and for some students the thought of waiting an additional year before graduating and beginning clinical practice will be unappealing. A year is also a long time to commit to spend studying a subject, so it is crucial that students have done their research and are motivated from the outset. At some UK medical schools intercalation is compulsory, so it is important that students think hard before applying to such a course about whether they are prepared to commit to completing an intercalated degree at that early stage ( Rushforth, 2004). In other medical schools where intercalation is optional, the decision to take a year away from the standard course will then place students out of sync with their peers. It is thus critical that students spend time weighing up the relative arguments for and against intercalation and reach their own personal decision.

### Tip 2: research potential supervisors carefully

A student’s experience during their intercalated research project is heavily influenced by their supervisor and their relationship with them ( Möller, Shoshan and Ponzer, 2015). It is very important that students spend time researching possible supervisors and the available projects. Wherever possible students should arrange to meet with potential supervisors to discuss project proposals and ultimately try to establish whether they can imagine themselves working well within a particular research group. It is also very helpful to speak to previous students who have worked in a particular laboratory to discuss their experiences and gain their personal insight into working with a certain supervisor. Another important consideration when exploring possible supervisors is to consider how research-active a particular supervisor is. Students are more likely to collaborate on meaningful research that may be published in highly research-active laboratories with ambitious supervisors ( Amgad *et al.*, 2015).

### Tip 3: choose a project in a genuine area of interest

The majority of the medical curriculum is very prescriptive, and thus the new-found freedom presented by an intercalated research project can be daunting with so many different opportunities on offer. In choosing an intercalated research project, it is crucial that students pick a project that really excites them, and they are not swayed by what others suggest they ‘should’ choose ( Yang *et al.*, 2019). Intercalated research projects represent a significant investment of time and effort, and completing the work to a high standard will be incredibly difficult if students lack a real interest in what they are studying. Intercalation also represents a valuable opportunity to truly explore a subject in detail, and by choosing to undertake a project in a field of interest, this can give medical students a fantastic opportunity to discover if this is an area they would like to pursue further in their future career ( Brown *et al.*, 2018).

### Tip 4: spend time getting to know the other members of the laboratory

As well as developing a good relationship with the project supervisor, it is equally important to get to know the other members of the laboratory. In reality, project students will often spend much more of their time with the laboratory’s post-doctoral researchers and technicians than their supervisor, and it is thus crucial to develop an excellent working relationship with these laboratory members ( Chang and Ramnanan, 2015). They can provide project students with vital advice on all aspects of their research work, which is particularly important during the early stages of a student’s time in the laboratory. Having a good relationship with all members of the research team is also vital for a project student to feel truly integrated within the laboratory and vastly improves the overall quality of their project experience.

### Tip 5: be pro-active and enthusiastic

Compared to other more didactic elements of medical education, what a student gets out of an intercalated research project is hugely dependent on the amount of effort they put in. If medical students demonstrate genuine enthusiasm for their laboratory work and pro-actively contribute to the planning and construction of a project, they will be able to both maximise the success of their project, as well as develop a wealth of new skills. This demonstration of commitment by a project student will in turn lead to their supervisor and other research group members being prepared to invest more time and energy into mentoring the student, leading to a virtuous cycle with optimal outcomes for all those involved in the work ( Hunskaar *et al.*, 2009).

### Tip 6: do not spend all of your time in the lab

Although it is crucial that students display a high level of dedication to their project work as outlined in tip 5, it is equally important that students manage their time effectively and appreciate that working ten hours every day in the laboratory is not needed to demonstrate enthusiasm ( Gordon and Borkan, 2014). Laboratory work is usually only one component of an intercalated degree programme, and it is critical that students do not neglect the other aspects of their course such as lectures, tutorials and supervisions. This balance can be difficult to strike, particularly during the early phase of a project where it is very easy to be drawn into spending increasingly longer periods in the laboratory to kickstart the work and make a good impression. Students must therefore develop excellent self-discipline and should not be afraid of speaking to their supervisor if they are concerned that other aspects of their study are suffering as a result of excessive demands of their practical work.

### Tip 7: do not be scared of asking for support

The commencement of an intercalated degree programme can be a very turbulent period, requiring students to adapt to a very new style of learning and working, and it is only natural for students to find things challenging ( Can *et al.*, 2016). An intercalated research project will be very different to the previous years of a medical student’s education and may be their first experience of practical laboratory work. As a result, medical students can find beginning a laboratory-based project more challenging than peers conducting biological sciences degrees. Medical students should remember that supervisors will be aware of these difficulties and will be fully prepared to provide them with the necessary support. It is far healthier that students discuss concerns at an early stage so that any issues can be swiftly addressed and that potential problems do not progress into anything more serious. Supervisors also need to be able to trust students to work safely within the laboratory, and it is therefore important that if students are unsure how to carry out a particular task they ask for help. Other laboratory members will be aware that many aspects of research work will be unfamiliar to project students and will be happy to provide support so students should not be apprehensive of reaching out to them.

### Tip 8: stay positive

Practical science can be unpredictable, with experiments frequently not generating the results that were hoped for. It can be incredibly disheartening for students when their project work does not go as anticipated, requiring them to repeat or redesign experiments. This is often acutely felt by project students who only have a relative short period of time in order to complete their practical work. It is thus crucial students develop a strong sense of resilience at an early stage and are not put off if experiments are initially unsuccessful. Project students should also remember that the grade awarded to their project work does not hinge on the results that they generate, and that very good project reports can be written even with minimal data. Some of the best marks may be awarded to students who have less data but are able to insightfully and maturely reflect on the challenges they faced and make suggestions for future work to be conducted ( Brown, Lewis and Bevan, 2016).

### Tip 9: start writing up your project early

The final writing up of a research project is often something that strikes fear into the hearts of students, and it can be very tempting to put off beginning this process until the deadline is fast approaching. The write up of an intercalated research project will frequently be a medical student’s first piece of scientific writing, and thus the unfamiliarity of the style of writing required can make the process particularly challenging ( Hyatt, Bienenstock and Tilan, 2017). It is therefore crucial that students begin writing their projects up as early as possible, as this facilitates ample time for input from their supervisor and multiple revisions to be drafted ( Singh and Mayer, 2014). Many universities will have archives of the reports of past project students, and so by beginning to think about writing up their project early, students will have sufficient time to carefully review the work of past students to help inform them as to the style and structure of writing expected from them.

### Tip 10: do not become fixated on the report word count

The majority of project write up submissions will have a maximum word count that students must adhere to. It is easy for project students to become fixated on this number from the outset, and write their report focused on meeting the word count restrictions, as opposed to actually producing a comprehensive report that fulfils the marking criteria. Students should not focus on the word count initially but instead write freely, conveying everything they would like to include in their report. It is far easier to edit a report that is too long in order to meet the word count, than to manipulate in extra text at the last minute into a report that is too short ( Iskander *et al.*, 2018).

### Tip 11: seek out chances to present your work

The skills that students acquire from undertaking an intercalated research project extend beyond those developed in the laboratory. Having completed their project work, medical students should seek opportunities to present their work. It is usually straightforward to present findings at the departmental level via the project supervisor, and in some instances, it may be a mandatory part of the student’s project assessment. Many medical schools also run conferences throughout the year, allowing students to deliver oral and/or poster presentations on their research. Depending on the outcomes of projects, it may be possible for medical students to present their work at regional or national conferences ( Griffin and Hindocha, 2011). Presenting project work is a valuable experience for medical students, being a sought-after skill of both clinicians and academics alike ( Ridde and Mohindra, 2009). Honing these skills early on in their medical education will be hugely beneficial as they progress onwards throughout their career.

### Tip 12: keep in touch with your supervisor

On completing their laboratory work, students often loose contact with their supervisor. However, by staying in contact with project supervisors beyond the end of their initial project, students can remain engaged with the research group and get involved in further collaborations in the future ( Dyrbye, Davidson and Cook, 2008). This can be of great benefit in enabling students to develop an even deeper insight into the research field and is a great way to demonstrate enthusiasm for a particular speciality if students are considering pursuing a career in that area. Critically, if the student’s project work is going to be part of a paper that the laboratory is planning to submit for publication, it is crucial that students keep in touch so they can be actively involved in reviewing the manuscript, as well as critically ensuring they receive the author recognition they deserve. An intercalated research project presents a great opportunity for medical students to gain a publication in a peer-reviewed journal, which can markedly enhance future training programme applications ( Al-Busaidi, Wells and Wilkinson, 2019).

## Conclusion

Laboratory-based intercalated research projects are commonly carried out by medical students and offer many opportunities to acquire new skills and enhance a student’s portfolio. They are however not an easy undertaking, which is why the successful completion of an intercalated research project is regarded so highly. Students often face a steep learning curve at the beginning of a research project, with lots for them to learn regarding how to approach and manage their workload in a very short time. It is therefore recommended that medical students review the twelve tips outlined in this article in order to ease the transition into their laboratory work and maximise their project outcomes.

## Take Home Messages

• Laboratory-based intercalated research projects enable medical students to engage in academic research during their undergraduate studies
• Completing a laboratory-based intercalated research project can be both challenging and rewarding
• Thoroughly preparing before choosing an intercalated research project can help medical students enhance their experience
• High levels of commitment, enthusiasm and organisation during the project can optimise the project outcomes

## Notes On Contributors

Lydia E. Roberts, BA, is a clinical medical student who completed an intercalated research project in the Department of Pathology, University of Cambridge. ORCID iD: https://orcid.org/0000-0002-3754-3389
