# Supplementary material for: Twelve tips for UK medical students undertaking laboratory-based intercalated research projects
Source: MedEdPublish (2016). 2021 Sep 3;9:225. Originally published 2020 Oct 8. [Version 2] doi: 10.15694/mep.2020.000225.2 (PMC10448456; doi:10.15694/mep.2020.000225.2)
Supplement: Peer review history

# Reviewer report: Reviewer response for version 2

**DOI:** 10.21956/mep.20283.r31491

**Published:** 2021-09-08

**Authors:** Ken Masters

**Affiliations:** 1 Sultan Qaboos University

This review has been migrated. The reviewer awarded 4 stars out of 5 This is a nicely-improved 12-tips paper (although submitted as a personal opinion piece), and the author has addressed my concerns about Version 1 (which were mainly on ensuring the geographical context and the focus on laboratory-based research was indicated in the Title and Abstract.)The paper will be useful for students and supervisors in the UK; students and supervisors from other areas should also be able to identify pertinent principles applicable to their own contexts. Nicely done.

Reviewer Expertise:

NA

No decision status is available

# Reviewer report: Reviewer response for version 2

**DOI:** 10.21956/mep.20283.r31492

**Published:** 2021-09-06

**Authors:** Simonetta Ausoni

**Affiliations:** 1 Department of Biomedical Sciences- University of Padova- School of Medicine

This review has been migrated. The reviewer awarded 5 stars out of 5 This is a very interesting work that I have read with great pleasure. The manuscript is aimed at medical students who want to undertake an intercalated research project, and offers them multiple points of reflection on the experience of intercalation. I recommend reading it, not only to students, but also to teachers of medical courses who have not yet adopted this path, but may still be interested in expanding the laboratory experience of medical students. In adding the notes to the revision (page 1) the author omitted the punctuation. It would be useful to fix it in print, perhaps without sending the work back to the author.

Reviewer Expertise:

NA

No decision status is available

# Reviewer report: Reviewer response for version 2

**DOI:** 10.21956/mep.20283.r31493

**Published:** 2021-09-04

**Authors:** Neslihan Onder-Ozdemir

**Affiliations:** 1 The University of Sheffield

This review has been migrated. The reviewer awarded 5 stars out of 5 Thank you for your submission. In the Introduction section, the intercalated degree programme in medical education has been explained with a critical eye through the discussion of its advantages and disadvantages. In light of the present literature, the author’s personal experience of conducting an intercalated research project as a clinical medical student who completed an intercalated research project in the Department of Pathology in the UK is also insightful given that this is the first-hand experience. This personal view can help prospective students and also medical schools may benefit from this experience that has been presented in light of literature. Some editing recommendations:In the Introduction subsection1- in the first paragraph, which is below, after "however" and "frequently", we can put a comma"... their choosing, however most frequently students..."2- In the fourth paragraph, before and after the verb "come," we need "to.""This can leave students frustrated come the..." 3- Under Tip 7, please remove "to" after the adjective "different" and put "from": should be different from. Please combine "may" and "be", which should be "maybe.""An intercalated research project will be very different to the previous years of a medical student’s education and may be their first experience of practical laboratory work."4- Please put a dash between the words "Take" and "home": Take-home messages

Reviewer Expertise:

NA

No decision status is available

# Reviewer report: Reviewer response for version 1

**DOI:** 10.21956/mep.19012.r27282

**Published:** 2021-01-10

**Authors:** Ken Masters

**Affiliations:** 1 Sultan Qaboos University

This review has been migrated. The reviewer awarded 3 stars out of 5 A useful 12-tips paper for medical students considering taking an intercalated research year. There are several things that I like about this paper, not least is the balanced view of the year. While it is apparent that the author found the year beneficial, they also balanced their enthusiasm with responsible and realistic considerations for the workload and demands, and the impact of such a year.Overall, the paper also has a good balance of personal experience and drawing on the literature.Two points that I would like to see addressed in Version 2 of this paper:• As has been pointed out by another reviewer, the context is the UK. As the structure of medical degrees differs substantially across the world, and this would influence many aspects of the intercalated choices, I would recommend that both the title and the abstract make it clear that this applies to the UK only. (Many other countries will benefit from the paper, but readers need to realise that the context may be different).• The focus of the research in this paper is on laboratory work. That may be because of the author’s personal experience. There is, however, a wide range of research projects (e.g. EMR analysis, the effectiveness of AI systems, surveys, etc.) that do not involve laboratory work. So, either the title and abstract (and limitations) should reflect this, or aspects of the paper could be expanded a little. Overall, a useful paper, and I look forward to Version 2 in which these issues are addressed.

Reviewer Expertise:

NA

No decision status is available

# Reviewer report: Reviewer response for version 1

**DOI:** 10.21956/mep.19012.r27283

**Published:** 2020-12-24

**Authors:** Caroline Bowsher

This review has been migrated. The reviewer awarded 4 stars out of 5 Overall this is an interesting article providing a useful insight to undertaking a research project as part of an intercalated degree. The 12 tips provided are useful and link well with available literature from the field. Interestingly the advice is useful beyond the stated audience and highlights many of the challenges a new student encounters when they begin a research project. As such this article will be one I recommend to any new research project student. Perhaps an additional consideration would have been to flag further the unique challenges a medical student might experience over those 'other' students undertaking a research project as it is these areas which a supervisor might benefit from being reminded of to further support the initial transition of undertaking a research project.

Reviewer Expertise:

NA

No decision status is available

# Reviewer report: Reviewer response for version 1

**DOI:** 10.21956/mep.19012.r27281

**Published:** 2020-11-12

**Authors:** Barbara Jennings

**Affiliations:** 1 Norwich Medical School

This review has been migrated. The reviewer awarded 3 stars out of 5 Thank you to the author for a very well written and interesting analysis of the advantages and challenges of undertaking an intercalated degree during undergraduate medical studies. I will certainly be recommending this article to students considering similar projects and to the tutors who advise them on these options.There are lots of possible conflicts of interest for institutions recommending intercalated degree years (that are costly to the learner in time and money) and I am concerned that cultural capital can be a significant confounding factor when the favourable impact of intercalated research degrees is presented. I have been a supervisor for short research degree programmes and an academic advisor to medical students for many years and I never recommend intercalation without some reservation, and without pointing out many caveats. It was refreshing to read such a full and well-researched analysis from a learner’s perspective – noting both the career and scholarly advantages as well as some potential pitfalls.In the introduction and throughout the twelve tips, the author cited interesting commentaries on the subject of intercalation. Research studies were also cited and integrated well, including a useful systematic review and meta-analysis from 2015 (https://doi.org/10.1371/journal.pone.0127470).My only reservations relate to an inconsistency with one of the citation formats in the bibliography (a very minor point) and the generalisability of the article. The author explains that it reflects personal experience as well as an appraisal of the literature, and this individual perspective makes it engaging but most relevant to particular research carried out in the context of a UK medical career (i.e. the concrete advantages to medical graduates with their first job applications and the emphasis on laboratory studies). I wondered if an opinion/personal perspective article (rather than a guidelines article) might have been more appropriate because of this and because it is a single-author article? Many medical undergraduates select clinical research degrees and spend considerable time learning about, and grappling with, research governance and so in that context the top tips might be quite different. However, the reflection and sage advice in each of the tips are certainly worth reading and sharing.

Reviewer Expertise:

NA

No decision status is available
